# Analysis of Single Particles of Amyloid Beta and α‐Synuclein With Seeded Amplification for the Diagnosis of Alzheimer's and Parkinson's Disease

**DOI:** 10.1002/bab.70083

**Published:** 2025-10-31

**Authors:** Alexandra Dybala, Marlene Pils, Oliver Bannach, Gültekin Tamgüney, Detlev Riesner

**Affiliations:** ^1^ Mathematisch‐Naturwissenschaftliche Fakultät, Institut für Physikalische Biologie Heinrich‐Heine‐Universität Düsseldorf Düsseldorf Germany; ^2^ attyloid GmbH Düsseldorf Germany; ^3^ Institute of Biological Information Processing (Structural Biochemistry: IBI‐7) Forschungszentrum Jülich Jülich Germany

**Keywords:** aggregation, biomarker, neurodegenerative diseases, pathology, seeds, surface‐based fluorescence intensity distribution analysis (sFIDA)

## Abstract

Neurodegenerative diseases, including Alzheimer's disease (AD) and Parkinson's disease (PD), are characterized by the pathological aggregation of specific proteins such as amyloid beta (Aβ) and α‐synuclein, respectively. Early detection of these protein aggregates in biological fluids could facilitate timely diagnosis and therapeutic intervention. This study explores an aggregate amplification approach using the surface‐based fluorescence intensity distribution analysis (sFIDA) method to enhance the detection sensitivity of Aβ and α‐synuclein seeds. Two amplification strategies were investigated: surface‐bound and solution‐phase amplification. In the case of Aβ, surface‐bound amplification using immobilized Aβ‐specific antibodies was tested with synthetic Aβ_1‐42_ seeds and monomeric Aβ_1‐42_ as the substrate. Despite observable aggregation, self‐aggregation of the monomeric substrate interfered with seed‐dependent amplification, rendering the approach ineffective at physiologically relevant concentrations. Attempts to suppress self‐aggregation using blocking peptides, bovine serum albumin, and truncated Aβ_11‐42_ substrates were unsuccessful. Solution‐phase amplification followed by surface detection also failed to reliably differentiate seeded aggregation from self‐aggregation, indicating that Aβ amplification is unsuitable for diagnostic applications. In contrast, α‐synuclein exhibited significantly lower self‐aggregation, allowing for more effective seeded amplification. Surface‐bound α‐synuclein amplification successfully detected synthetic seeds at nanomolar concentrations, while solution‐phase amplification further improved sensitivity, enabling detection down to the picomolar range. The method was successfully applied to brain homogenates from transgenic PD model mice, demonstrating the potential for detecting α‐synuclein seeds in biological samples. These findings highlight the limitations of Aβ amplification for diagnostic purposes while supporting the feasibility of α‐synuclein amplification for PD detection. Future work will focus on optimizing this approach for clinical applications.

**Abbreviations**
AD: Alzheimer's disease
Aβ: amyloid beta
BSA: bovine serum albumin
PBS: phosphate‐buffered saline
PD: Parkinson's disease
PTA: sodium phosphotungstate
Sfida: surface‐based fluorescence intensity distribution analysis
TBS: tris‐buffered saline
TBSP: TBS with 0.03% Proclin 300
TBST: TBS with 0.01% Tween 20
ThT: thioflavin T

## 1 Introduction

Neurodegenerative diseases are often characterized by the pathological aggregation of cellular peptides or proteins [1]. Among these predominantly age‐related disorders, Alzheimer's disease (AD) is the most prevalent [2]. AD is marked by the aggregation of amyloid beta (Aβ) peptides and tau proteins, whereas the aggregation of α‐synuclein is a hallmark of Parkinson's disease (PD), the second most common neurodegenerative disorder [3, 4, 5, 6, 7, 8]. These aggregated proteins can accumulate in the brain and other tissues or spread through the body as smaller oligomers, which can be detected in cerebrospinal fluid and, at very low concentrations, in blood [9, 10, 11, 12, 13, 14, 15, 16].

While detecting disease‐specific oligomers in blood would be an ideal biomarker, it is not yet routinely feasible [15]. An advanced test does exist for detecting AD‐specific tau phosphorylated at amino acid 217 in plasma [17, 18]. Recently approved treatments with specially developed antibodies targeting Aβ, lecanumab and donanemab, have shown some ability to slow mental decline, but no definitive cure for AD or PD has been achieved [19, 20]. The prospects for slowing or curing these diseases would be significantly enhanced by early diagnosis.

To detect low concentrations of biomarkers in blood, the highly sensitive surface‐based fluorescence intensity distribution analysis (sFIDA) method has been developed [12, 13, 14, 21, 22, 23, 24, 25, 26]. This technique is used to identify disease‐related aggregates of the prion proteins, Aβ, tau, and α‐synuclein. Detection sensitivity may be further enhanced by adding monomers to disease‐associated oligomers, which could increase the surface area of protein aggregates and expose more epitopes for detection probes.

Here, we tested various parameters for the amplification and detection of Aβ and α‐synuclein aggregates. A key advancement was the transition from amplifying immobilized aggregates on a surface to amplifying them in solution, followed by their binding to surface‐anchored antibodies and detection using one or two fluorescence‐labeled antibodies. Promising results were achieved with α‐synuclein aggregates extracted from brain homogenates of transgenic mice using an established protein precipitation method. Moving forward, this amplification and detection protocol for α‐synuclein aggregates will be optimized for application to biological samples such as cerebrospinal fluid or blood.

## 2 Materials and Methods

### 2.1 Surface‐Based Fluorescence Intensity Distribution Analysis (sFIDA)

The sFIDA assay was conducted in 384‐well microtiter plates (Nunc MicroWell 384‐Well Plates, Thermo Fisher Scientific, or Sensorplate plus 384 Well, Greiner Bio‐One International). Washing steps were performed automatically using a 405TS microplate washer (BioTek Instruments). The assay setup involved immobilizing capture antibodies on the glass surface in 0.1 M carbonate buffer at 4°C overnight. We used the NAB228 (Sigma‐Aldrich) or 6E10 (BioLegend) antibodies at 2.5 µg/mL to capture Aβ, and the Syn211 antibody (Santa Cruz) at 7.5 µg/mL to capture α‐synuclein [27, 28, 29]. Preparation steps were performed at room temperature unless stated otherwise. Following five wash steps with tris‐buffered saline (TBS) with 0.01% Tween 20 (AppliChem; TBST), unbound surfaces were blocked with TBS pH 7.4 with 0.03% Proclin 300 (TBSP; Sigma‐Aldrich) and 0.5% bovine serum albumin (BSA, AppliChem) for 1.5 h, followed by another wash step as described above. Amplification of α‐synuclein was performed in 40 or 50 mM tris buffer pH 8.0 and 0.03% Proclin 300 at 37°C. Incubation was followed by five wash steps with TBS. Detection antibodies, if used, were bound to the target proteins for 1 h, followed by five wash steps with TBS. A buffer exchange with TBSP was performed to prevent contamination. The plate was sealed with SealPlate film (Sigma‐Aldrich) and measured.

For the preparation of detection antibodies, the Syn211 or 4D6 (BioLegend) antibodies for α‐synuclein were conjugated with the fluorophores CF633 or CF488A (Sigma‐Aldrich) [30, 31]. These dyes contain succinimidyl esters, which react with primary amines of antibodies under basic conditions. The conjugation reaction was conducted in a 1 M carbonate solution for 1 h at room temperature while shaking at 600 rpm in a Thermomixer Comfort (Eppendorf). Fluorophore‐conjugated antibodies were purified using a polyacrylamide matrix (Bio‐Gel P‐30 Gel, Bio‐Rad Laboratories) to remove excess dye. To remove aggregates from detection antibodies or substrate solutions, the prepared solutions were ultracentrifuged for 1 h at 100,000 × *g* at 4°C using an Optima Max‐XP ultracentrifuge (Beckman Colter) equipped with a TLA 100.3 fixed‐angle rotor (Beckman Colter). The supernatant was carefully transferred to a fresh reaction tube. Antibody concentration was subsequently determined photometrically using a V‐650 UV/Vis spectrophotometer (Jasco). The Syn211 and 4D6 antibodies were used at 0.625 µg/mL, and the at NAB228 antibody 0.31 µg/mL.

Fluorescence intensity measurements were performed using a Celldiscoverer 7 microscope (Zeiss Jena AG) equipped with an Evolve camera (Thermo Fisher Scientific). Two fluorescence channels were used: excitation wavelength (*λ*_Ex_) 653 nm and emission wavelength (*λ*_Em_) 668 nm for the red channel, and *λ*_Ex_ 493 nm and *λ*_Em_ 517 nm for the green channel. Emission wavelengths passed through either a 670–710 nm or 504–554 nm filter. Plan‐Apochromat objectives (50×/1.2 or 20×/0.7) were used for 50× or 20× magnification, respectively, with an optional additional lens for doubled magnification.

Samples were measured in quadruplicate in 384‐well microtiter plates. For each well, a 5 × 5 image matrix was captured, providing a total of 100 images per condition for analysis. The sFIDAta software was used for image analysis, which included automatic artifact detection to exclude blurred images, images with particulate contamination, or images deviating by more than 250% from the mean brightness. Average pixel intensities were computed for each well, and the mean of four replicates was determined. The resulting intensity distribution was visualized as a histogram. Each image contained 512 × 512 pixels (262,144 total pixels) with 65,536 possible grayscale values. The pixel count was plotted against intensity values, and cumulative pixel counts were calculated for intensities exceeding a defined cutoff value. The cutoff was determined based on background noise, tolerating 0.1% of the brightest pixels (262 pixels out of 262,144). Pixels were classified as above or below this threshold, and the pixel count above the cutoff was displayed as a bar graph with standard deviation from four replicates.

### 2.2 Aβ Aliquoting

Lyophilized Aβ_1‐42_ or truncated variants (1 mg; Bachem) were dissolved in 1100 µL of 1,1,1,3,3,3‐hexafluoro‐2‐propanol (Sigma‐Aldrich) and incubated for 10 min at room temperature. The solution was then aliquoted into 50 µg portions (50 µL each) using a microliter syringe (Hamilton) into 1.5 mL tubes. Aliquots were snap‐frozen in liquid nitrogen and lyophilized using a RVC 2‐18 vacuum centrifuge (Martin Christ).

### 2.3 Thioflavin T Aggregation Assay

Experiments were conducted in a 96‐well microtiter plate (Half Area, non‐binding surface, Corning) with a total volume of 100 µL per well. The Thioflavin T (ThT; Eurogentec)/protein ratio was approximately 1:3, with a minimum ThT concentration of 3 µM for low protein concentrations. Fluorescence measurements were performed using a FLUOstar Omega or CLARIOstar plate reader (BMG Labtech) with an excitation filter of 440 nm and an emission filter of 480 nm. Data points were collected at intervals of 3 to 6 min. Measurements were taken without shaking in a 20 mM Tris buffer pH 8.0 with 0.03% Proclin 300 at 25°C for Aβ, and in a 50 mM Tris buffer pH 7.5 with 0.03% Proclin 300 at 37°C for α‐synuclein. For monomeric Aβ, lyophilized aliquots were dissolved in 10 mM NaOH on ice before mixing with buffer solutions containing ThT, sodium azide, and other reagents. Samples were rapidly transferred into the wells of the microtiter plate. For amplification assays, seeds were first added to the wells before the substrate solution was introduced. The substrate solution was kept on ice. Assays with α‐synuclein followed the same procedure as Aβ assays.

### 2.4 α‐Synuclein Protein Synthesis and Purification

The transformation of the plasmid vector encoding either human wildtype α‐synuclein or its Y136C mutant (Y136C‐α‐Syn) was performed by adding 1 µL of a 20 ng/µL plasmid vector solution to 50 µL of chemically competent *Escherichia coli* XL1‐Blue cells. The mixture was incubated on ice for 10 min, followed by a heat shock at 42°C for 45 s. The reaction was then placed back on ice for 2 min and subsequently incubated in 500 µL LB medium (PanReac AppliChem ITW Reagents) at 37°C with shaking at 800 rpm for 30 min. The bacterial culture was plated onto an LB agar plate (AppliChem) containing 100 µg/mL ampicillin (AppliChem) and incubated overnight at 37°C. A single colony was used to inoculate a 50 mL overnight culture in 2YT medium (AppliChem) supplemented with 100 µg/mL ampicillin, which was incubated at 37°C with shaking at 160 rpm. Plasmid isolation was performed using the Nucleospin Plasmid EasyPure Kit (Macherey‐Nagel) following the manufacturer's protocol, and the isolated vectors were subsequently transformed into *E. coli* BL21 (DE3).

A subsequent overnight culture was prepared, and 2 L of bacterial culture was grown in a medium consisting of 31 g/L 2YT medium, 2 mM MgSO_4_, 50 mM phosphate buffer (37.4 mM Na_2_HPO_4_ + 12.6 mM NaH_2_PO_4_), and 100 µg/mL ampicillin. For autoinduction, the medium was supplemented with 0.2% lactose (Caesar & Loretz GmbH), 0.05% glucose (Caesar & Loretz GmbH), and 0.6% glycerol. The culture was inoculated with an optical density at 600 nm of 0.05 from the overnight culture and incubated under the same conditions for approximately 24 h. Cells were harvested by centrifugation at 5000 × *g* for 20 min. The bacterial pellet was resuspended in 0.9% NaCl solution and incubated with an EDTA‐free protease inhibitor cocktail (cOmplete, Roche) at a concentration of one tablet per 20 mL solution. The suspension was lysed thermolytically by incubation at 95°C for 15 min in a 50 mL reaction tube, with intermittent mixing. After cooling on ice, the lysate was centrifuged at 8000 × *g* and 4°C for 20 min.

The supernatant containing the target proteins was transferred to a fresh 50 mL reaction tube, and DNA was precipitated by adding streptomycin sulfate to a final concentration of 10 mg/mL, followed by mixing on a roller mixer at 4°C for 20 min. The sample was centrifuged again at 8000 × *g* and 4°C for 20 min. The supernatant was filtered through a 0.22 µm syringe filter (ROTILABO polyvinylidene fluoride, Carl Roth) into a fresh reaction tube, and proteins were precipitated using a saturated ammonium sulfate solution (VWR International). After centrifugation, the white protein pellet was either stored at −20°C or dialyzed overnight at 4°C in 25 mM Tris buffer (VWR International) at pH 8.0 using a 10 kDa molecular weight cutoff dialysis tube.

Anion exchange chromatography was performed using a HiTrap Q HP 5 mL column (Cytiva) in 25 mM tris, pH 8.0, with a salt gradient increasing up to 800 mM NaCl at a rate of 2% per minute and a flow rate of 2 mL/min. The protein was again precipitated with ammonium sulfate and prepared for size‐exclusion chromatography. WT‐α‐Syn was resuspended in 25 mM Tris pH 8.0, while Y136C‐α‐Syn was incubated for 1 h in 150 mM Tris pH 7.2 supplemented with 5 mM tris(2‐carboxyethyl)phosphine hydrochloride to reduce oxidized cysteines. Size‐exclusion chromatography was carried out using a Superdex 200 Increase 10/300 GL column (GE Healthcare) in the same buffer. Wild‐type α‐synucelin was eluted in 50 mM Tris pH 8.0 and stored at −80°C. The Y136C‐α‐Syn mutant was either shock‐frozen and stored at −20°C for short‐term use or immediately prepared for fluorophore labeling.

The protein concentration was determined photometrically using a V‐650 UV/Vis spectrophotometer at 275 nm with a molar extinction coefficient of 5600 M^−1^ cm^−1^ for α‐Syn‐WT and of 4110 M^−1^ cm^−1^ for Y136C‐α‐Syn.

### 2.5 Fluorochrome Conjugation of Y136C‐α‐Syn Mutant

The Y136C‐α‐Syn solution was centrifuged using a 10 kDa cutoff centrifugal filter at 3200 × *g* and 4°C. The supernatant was replenished to its original volume with 200 mM HEPES buffer. The solution was then transferred to a 1.5 mL reaction tube and incubated with a 5.4‐fold molar excess of Hilyte‐Fluor‐647‐C2 maleimide (Anaspec) or Hilyte‐Fluor‐488‐C2 maleimide (Anaspec) in the dark at room temperature for 1 h. Excess dye was removed via high‐performance liquid chromatography using a semi‐preparative column (Zorbax 9.4 × 250 mm, Agilent). The elution solution contained 22% acetonitrile with 0.1% trifluoroacetic acid, with an increasing acetonitrile concentration gradient while maintaining a constant trifluoroacetic acid concentration: 28 min, 22%–40% acetonitrile (elution); 2 min, 40%–50% AcN; and 5 min, 80% acetonitrile (washing step). The eluate was then flash‐frozen in liquid nitrogen and lyophilized using an RVC 2‐18 vacuum centrifuge.

### 2.6 Preparation of Seeds

Aβ seeds were prepared in a 96‐well multititer plate using phosphate‐buffered saline (PBS, Sigma‐Aldrich) with 0.07% sodium azide (Sigma‐Aldrich). We used either unlabeled Aβ_1‐42_, HiLyte‐Fluor‐488‐labeled Aβ_11‐42_ and Aβ_1‐42_ (Anaspec), and HiLyte‐Fluor‐647‐labeled Aβ_1‐42_ (Anaspec). A total of 100 µL of protein solution at 5 or 10 µM was added to each well, sealed with a film (Seal Plate film, Sigma‐Aldrich), and incubated overnight at 37°C. Before use, the seeds were sonicated for 10 min in a Sonorex RK100H ultrasonic bath (Bandelin). α‐Synuclein seeds were prepared at a concentration of 100 µM in PBS with 0.7% sodium azide in a 1.5 mL reaction tube and incubated at 600 rpm in a thermomixer at 37°C. For systematic optimization of the amplification process, stock solutions of Aβ and α‐synuclein seeds were prepared. Aβ seeds were incubated at 10 µM in 20 mM phosphate buffer (pH 7.4) with 0.03% Proclin 300 and 25 mM NaCl at room temperature in a 384‐well multititer plate (Microplate 384/V‐PP, Eppendorf) for 1 day. Aβ solutions were pipetted in different volumes (16 × 30 µL, 16 × 50 µL, and 16 × 100 µL) into wells, combined, and sonicated three times for 1 s using a Microprobe MS72 ultrasonic probe (Bandelin). Aliquots (20 µL) were stored at 4°C.

The diversity of α‐synuclein seeds was achieved using glass beads of different sizes (0.75–1.00 mm and 2.85–3.45 mm, Carl Roth). A 96‐well multititer plate was filled with 50 µL of 100 µM α‐synuclein in PBS with 0.03% Proclin 300 (Sigma‐Aldrich). One‐third of the samples were incubated with small or large glass beads, while another third was incubated without beads at 37°C and 600 rpm in a plate reader for 7 days. The solutions were pooled and sonicated three times for 1 s using an ultrasonic probe. The seeds were then diluted to 10 µM, flash‐frozen in liquid nitrogen, and stored in 20 µL aliquots at −80°C.

### 2.7 Protein Precipitation With Sodium Phosphotungstate

A total of 60 µL of 20% brain homogenate from TgM83^+/−^ mice was diluted with 240 µL PBS to a final concentration of 5% and centrifuged at 5000 × *g* for 5 min. A 50 µL aliquot was mixed 1:1 with sarkosyl and sodium phosphotungstate (PTA; Sigma‐Aldrich) to achieve final concentrations of 2% sarkosyl and 2% PTA in PBS. The solution was incubated at 37°C and 650 rpm in a thermomixer for 1 h. A subsequent centrifugation step was performed for 30 min at 14,000 × *g* at room temperature. The resulting pellet was resuspended in 100 µL of 2% PTA and 2% sarkosyl in PBS and incubated for an additional 30 min. The pellet was then resuspended in 250 µL of 0.2% sarkosyl in PBS and sonicated three times for 0.1 s each, with 1‐s pauses, at 10% amplitude using an ultrasonic probe. The calculated brain homogenate concentration was 1%.

## 3 Results

**FIGURE 1:**
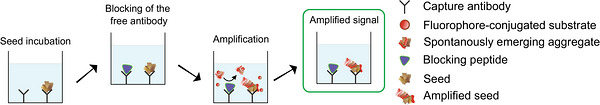
Schematic representation of the amplification process for seed‐oligomers bound to the surface. To mitigate false‐positive signals that may arise from the binding of spontaneously formed aggregates or fluorochrome‐conjugated substrate to the capture antibody, a blocking peptide is introduced after sample incubation, effectively blocking unbound capture antibodies. Subsequently, the substrate is added to amplify the seeds, while the blocking peptide prevents nonspecific binding of the substrate or newly formed aggregates. As a result, only the true positive signal from the amplified seeds is detected.

The assay's fundamental principle entails the amplification of Aβ‐ and α‐synuclein oligomers, which function as seeds, through the incorporation of Aβ‐ or α‐synuclein monomers, respectively. This process is followed by the detection of the resulting amplification using the sFIDA technique on a glass surface. Two distinct methodologies were employed. First, in surface‐bound amplification, the oligomer seeds were captured by specific antibodies that were immobilized on the surface. The resulting amplified aggregates were labeled with fluorescent antibodies and quantified using fluorescence microscopy. In the second approach, solution‐based amplification, the amplification process occurred in solution. The amplified aggregates were subsequently captured by surface‐bound, specific antibodies and detected using the same fluorescence microscopy technique as in the first method. Figure 1 illustrates the process of amplifying oligomeric seeds immobilized on a glass surface. To minimize the non‐specific binding of monomers, the unoccupied surface‐bound antibodies were blocked with a blocking peptide.

### 3.1 Amplification of Surface‐Bound Seeds

**FIGURE 2:**
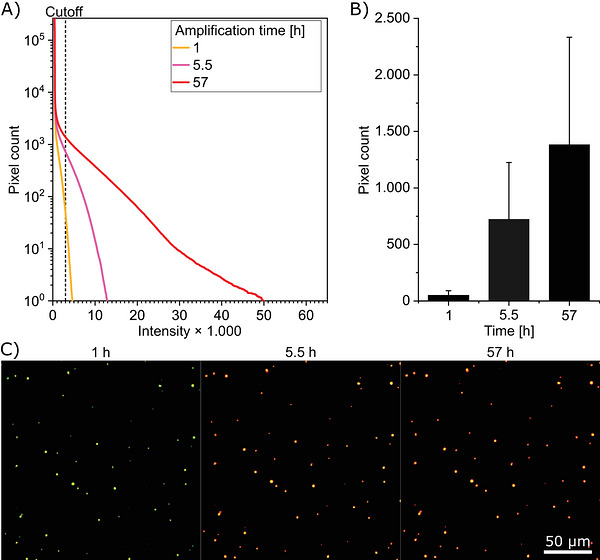
Imaging and quantification of the time‐dependent amplification of surface‐immobilized synthetic Aβ_1‐42_ seeds using sFIDA. In this experiment, 1 µM 10% HiLyte‐Fluor‐488‐labeled Aβ_1‐42_ seeds (green) were bound to the NAB228 capture antibody and incubated with 100 nM 10% HiLyte‐Fluor‐647‐labeled Aβ_1‐42_ monomer substrate (red). (A) Depicted are the integrated histograms of the signal (pixel count) measured in the red fluorescence channel after 1, 5.5, and 57 h of amplification. (B) A bar graph was used to represent the mean signal at a set cutoff of 3000 in the red fluorescence channel at 1, 5.5, and 57 h. Error bars represent the standard deviation. (C) Shown are exemplary images of Aβ_1‐42_ seeds (green) and Aβ_1‐42_ substrate (red), and their colocalization (yellow) after 1, 5.5, and 57 h.

The fundamentals of surface‐bound amplification were first demonstrated using 1 µM green‐labeled synthetic oligomeric Aβ_1‐42_ seeds, which were immobilized on a glass surface via capture antibodies. These were then incubated with 100 µM red‐labeled Aβ_1‐42_ monomers as the substrate. The fluorescence microscopy analysis revealed that the integrated number of red pixels with intensity exceeding the threshold increased over time (Figure 2). After 1 h of incubation, primarily green‐labeled oligomers were observed. Following 5.5 h, in addition to the green seeds, amplified products appeared as yellow pixels, indicating co‐localization of green and red signals. After 57 h of incubation, the fluorescence images displayed a greater number of pixels, predominantly yellow, reflecting significant amplification. In summary, the experiment successfully demonstrated amplification; however, the seed concentration of 1 µM remains relatively high and does not yet reflect the low concentrations of natural seeds. However, when the seed concentration was reduced to the nanomolar range, substrate self‐aggregation became predominant, effectively obscuring the seeded amplification. Various strategies to suppress Aβ peptide self‐aggregation using blocking peptides (Figure S1), BSA (Figure S2), and truncated Aβ_11‐42_ substrates (Supporting Information S1) were unsuccessful.

### 3.2 Amplification of Seeds in Solution

**FIGURE 3:**
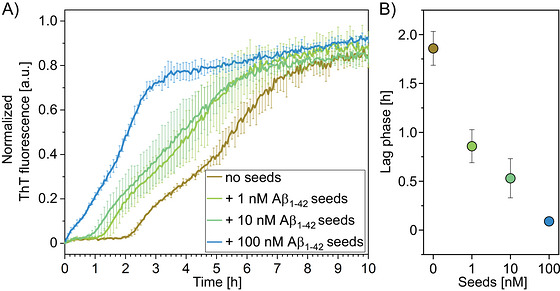
Increasing concentrations of Aβ_1‐42_ seeds accelerate the aggregation of Aβ_11‐42_ monomers in solution. (A) The Thioflavin (ThT) aggregation assay shows aggregation curves for 5 µM Aβ_11‐42_ measured normalized to a scale of 0 to 1, and subsequently averaged. Increasing Aβ_1‐42_ seed concentrations from 0 nM (brown) to 1 nM (dark green), 10 nM (light green), and 100 nM (blue) accelerated the aggregation of Aβ_11‐42_ monomers and resulted in concentration‐dependent reduction of the lag phase with a change from a sigmoidal to a linear amplification pattern. Aβ_11‐42_ was chosen to lower the tendency for self‐aggregation; this influence, however, was not significant. Data represent the mean values from triplicate experiments, with error bars indicating the standard deviation. (B) The mean lag phase of the aggregation curves was calculated for each seed concentration. Error bars represent the standard deviation.

Building on the observation that amplifying seed in solution prior to surface binding may offer advantages over direct surface‐based amplification, initial experiments were also conducted using Aβ seeds to explore this alternative approach. Using ThT fluorescence to monitor Aβ_11‐42_ aggregation, the experiments demonstrated a clear distinction between seeded amplification and self‐aggregation. Seeded amplification exhibited a linear profile, whereas self‐aggregation followed a sigmoidal pattern (Figure 3). While specific detection of 100 nM seeds was achievable, concentrations as low as 10 nM were obscured by self‐aggregation effects.

### 3.3 Amplification of α‐Synuclein Seeds

**FIGURE 4:**
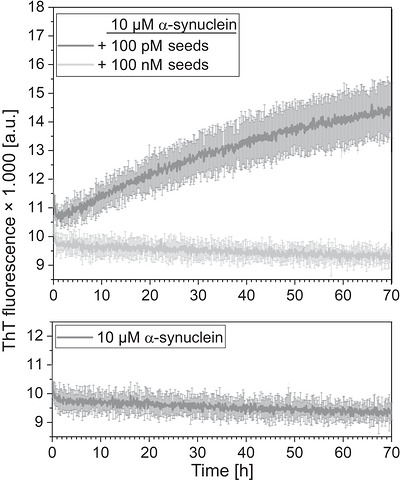
Assay optimization for α‐synuclein amplification in solution. Thioflavin T (ThT) fluorescence was used to monitor aggregation of 10 µM wildtype α‐synuclein monomers. Reactions were carried out using 100 pM (top panel, light grey), 100 nM (top panel, dark grey), or no α‐synuclein seeds (bottom panel, dark grey). Aggregation curves were measured in triplicate, averaged, and presented with error bars representing the standard deviation.

PD is associated with the aggregation of α‐synuclein, a 14 kDa protein. Unlike Aβ peptides, the self‐aggregation of α‐synuclein is significantly less pronounced [32]. Based on our experience with Aβ peptides, we hypothesized that the amplification system might perform more effectively with α‐synuclein. Similar procedures to those applied for Aβ peptides were implemented for α‐synuclein. In the initial experiment of an amplification in solution (Figure 4), 100 pM or 100 nM α‐synuclein seeds were incubated with 10 µM α‐synuclein substrate for up to 70 h. Amplification was monitored using ThT fluorescence. While 100 nM seeds were successfully amplified, significant amplification was not observed for 100 pM seeds. Importantly, self‐aggregation of the 10 µM substrate in the absence of seeds was not detected.

### 3.4 Amplification of Surface‐Bound α‐Synuclein Seeds

**FIGURE 5:**
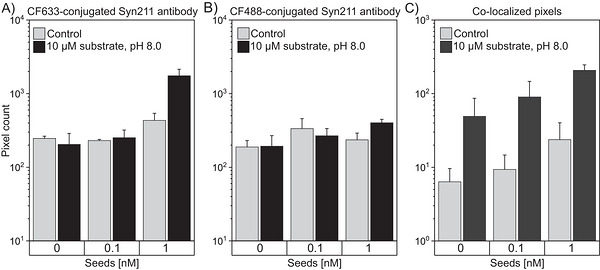
Amplification of surface‐immobilized α‐synuclein seeds. (A–C) Bar graphs depicting signal intensities after unseeded and seeded incubation for 20 h of no (control) or 10 µM α‐synuclein monomers. Fluorescence signals were measured using CF633‐conjugated Syn211 antibody (A) and CF488A‐conjugated Syn211 antibody (B), with co‐localization analysis shown in (C). The cutoff value was set at 0.1%. Error bars indicate standard deviation.

To enhance the sensitivity of seed detection, seeds were captured on surface‐bound Syn211 antibodies and incubated for 20 h with 10 µM substrate and visualized using CF633‐conjugated Syn211 antibody and CF488‐conjugated Syn211 antibody (Figure 5). While signals resulting from self‐aggregation were detected, a slight increase in colocalized particles was observed for 0.1 nM seeds, and a significant ∼4‐fold increase was observed for 1 nM seeds.

### 3.5 Amplification of α‐Synuclein Seeds in Solution

**FIGURE 6:**
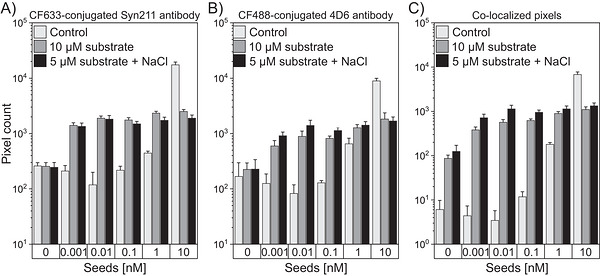
Amplification of α‐synuclein seeds in solution using either 10 µM substrate or 5 µM substrate with 12.5 mM NaCl. (A–C) Bar graphs showing fluorescence signals detected with CF633‐conjugated Syn211 antibody (A), CF488A‐conjugated 4D6 antibody (B), and their co‐localization (C). α‐Synuclein seed concentrations ranged from 0 to 10 nM. Substrate conditions included no α‐synuclein (control), 10 µM α‐synuclein without NaCl, and 5 µM α‐synuclein with 12.5 mM NaCl. The cutoff value was set at 0.1%. Error bars represent standard deviation.

Due to a substantial substrate background observed during surface‐based seed amplification in the absence of seeds, amplification was instead carried out in solution, followed by immobilization of the amplified seeds onto surface‐bound Syn211 capture antibodies. Detection was carried out using CF633‐conjugated Syn211 antibodies for red fluorescence and CF488‐conjugated 4D6 antibodies for green fluorescence (Figure 6). Despite the persistent presence of background substrate aggregates, 1 pM seeds produced a signal five times higher than the background, with 5 µM substrate proving slightly more effective than 10 µM. For seed concentrations exceeding 1 nM, direct detection without substrate amplification was more reliable (confer 1 and 10 nM seeds). Optimization of experimental conditions for the amplification of α‐synuclein seeds in solution, including substrate concentration, pH, salt concentration, and temperature, led to the identification of the following parameters: 5 µM substrate, pH 8.0, 12.5 mM NaCl, and 37°C.

In summary, α‐synuclein seeds are more readily detectable than Aβ seeds, likely due to the significantly lower self‐aggregation of α‐synuclein. Amplification in solution proved more effective than surface‐based amplification, and the use of fluorescence‐labeled detection antibodies demonstrated superiority over fluorescence‐labeled substrates for signal detection.

### 3.6 Detection of α‐Synuclein Seeds in Brain Homogenate From Sick TgM83^+/−^ Mice

Following the optimization of procedures using synthetic α‐synuclein seeds, we sought to detect seeds in brain homogenates from an animal model of PD. Brain homogenate from healthy and sick transgenic TgM83^+/−^, which expresses human A53T‐mutated α‐synuclein associated with familial PD, was used for this purpose [33]. In a previous experiment, these mice were injected intracerebrally with 50 µg of human wild‐type α‐synuclein fibrils to induce a PD‐like pathology, while control mice received 50 µg of BSA. Mice injected with α‐synuclein fibrils exhibited disease symptoms and had a median survival time of 133 days. Post‐mortem analysis revealed the presence of α‐synuclein fibrils and phosphorylated α‐synuclein in brain tissue sections. In contrast, control mice injected with BSA remained asymptomatic and survived beyond 500 days.

**FIGURE 7:**
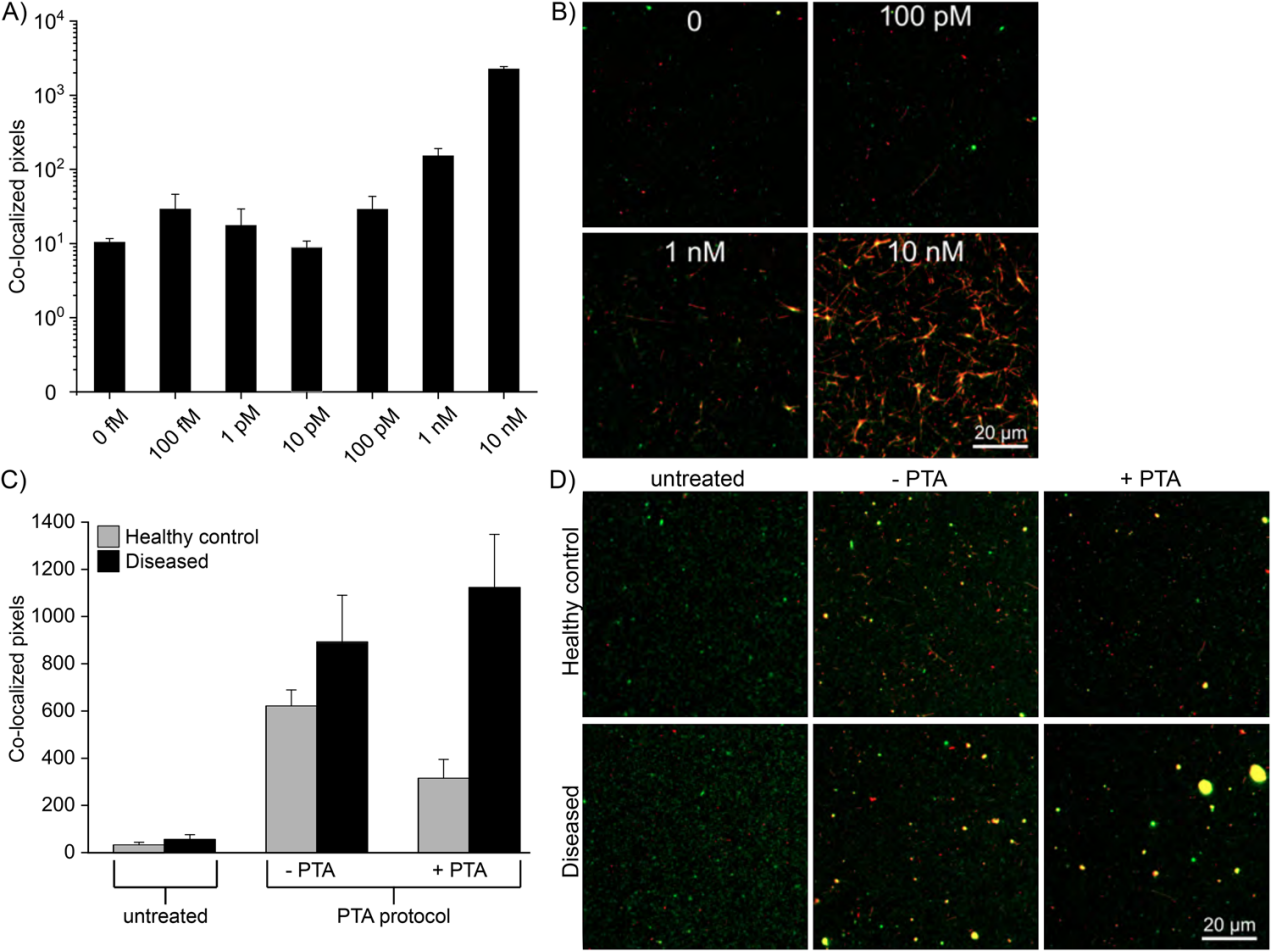
Amplification of surface‐immobilized α‐synuclein seeds. (A–D) Amplification of surface‐immobilized α‐synuclein seeds was performed for 4 days with 10 µM α‐synuclein monomer as substrate, 1% of which was Y136C‐α‐synuclein conjugated with Hilyte‐Fluor‐488 or with Hilyte‐Fluor‐647. (A) Bar graph showing co‐localized fluorescence signals of synthetic α‐synuclein seed concentrations from 0 to 10 nM. Signals increase from a seed concentration of 100 pM onward. (B) Example images of the concentrations of 100 pM–10 nM seeds and the substrate control without seeds (0). (C) Bar graphs showing co‐localized fluorescence signals corresponding to α‐synuclein aggregate levels in brain homogenates from healthy control (gray) and diseased TgM83^+/−^ mice (black) following exposure to 50 µg of either bovine serum albumin or wild‐type α‐synuclein fibrils. Homogenates were analyzed either untreated or processed using the phosphotungstic acid (PTA) precipitation protocol, which selectively precipitates α‐synuclein fibrils. To validate the efficacy of PTA precipitation, samples were assessed in the absence (−PTA) and presence (+PTA) of PTA. (D) Example images of the amplified brain homogenates from (C). Only co‐localized signals are shown in the bar graphs and in the images. The 0.1% cutoff was determined in the absence of seeds and the presence of substrate. Error bars represent standard deviation.

As a control experiment, synthetic α‐synuclein seeds of known concentrations were amplified on a surface using a substrate fluorescently labeled with either Hilyte‐Fluor‐488 or Hilyte‐Fluor‐647. The samples were incubated for 4 days at 37°C and pH 8.0. This amplification method enabled the detection of seeds at concentrations of 100 pM and higher (Figure 7).

The procedure was subsequently applied to a solution containing 1% brain homogenate from either diseased or healthy TgM83^+/−^ mice. To enhance the detection of α‐synuclein seeds, a seed concentration step using phosphotungstic acid (PTA) was implemented, which specifically precipitates fibrillar structures [34]. These steps significantly increased the concentration of α‐synuclein seeds (Figure 7). Without PTA precipitation, only minimal signals were detected, with no significant difference between healthy and diseased animals. Following the PTA precipitation protocol without actually adding PTA, an increase in signal was observed for both groups, with samples from diseased animals showing a 1.3‐fold higher signal. Following the full PTA precipitation protocol in the presence of PTA, a clear distinction between healthy and diseased animals was achieved, with a 3.5‐fold higher signal in samples from diseased animals.

## 4 Discussion

AD and PD are neurodegenerative disorders strongly associated with fibrillar forms of Aβ and α‐synuclein, respectively [35, 36, 37]. While effective therapies are not yet available for either condition, early detection can significantly benefit patients, and companion diagnostics are critical for therapeutic development [38, 39]. Both diseases involve a pathological transition of the respective proteins from monomeric cellular forms to aggregated fibrillar forms through a seeded aggregation process [16, 40]. This study aimed to exploit this mechanism by using fibrillar aggregates as seeds for further addition of fluorescently labeled monomers, thereby enlarging the aggregates and enhancing detection sensitivity. Detection was performed using sFIDA, which enables particle counting through fluorescence microscopy.

### 4.1 Seeded Aggregation and Self‐Aggregation in the Aβ System

To optimize the detection method, synthetic Aβ_1‐42_ seeds were immobilized on glass microscope plates via specific capture antibodies. Amplification was achieved by prolonged incubation with synthetic Aβ_1‐42_ peptides, allowing for the formation of enlarged fibrils. These were subsequently visualized using two detection antibodies, both specific to Aβ_1‐42_, conjugated to distinct fluorophores—one green and the other red. Our studies on the Aβ system revealed that seeded and self‐aggregation are competing mechanisms of the Aβ_1‐42_ peptide.

An experiment using 1 µM seeds and 100 nM substrate demonstrated that amplification increased linearly with incubation time. This is typical for seeded amplification. However, sigmoidal time dependence was observed at lower seed concentrations, indicating that Aβ_1‐42_ and truncated Aβ peptides strongly tend to self‐aggregate [41, 42].

In a diagnostic context, the seed concentrations are significantly lower, in the picomolar range or below. Under these conditions, self‐aggregation of the substrate dominated seeded aggregation. Several attempts were made to suppress self‐aggregation. These attempts included using blocking peptide Aβ_1‐14_ or BSA as a blocking agent; substituting Aβ_11‐42_ as the substrate, which binds to Aβ_1‐42_ seeds but not to the capture antibodies on the glass surface; and performing amplification in solution. However, none of these methods sufficiently differentiated seeded amplification from self‐aggregation. None of these attempts was successful.

Consequently, the seeded aggregation approach is not suitable for AD diagnostics. To improve sensitivity, particularly at diagnostically relevant low seed concentrations, future work could focus on strategies to reduce substrate self‐aggregation. It is conceivable that Aβ_1‐40_ and other N‐ and/or C‐terminally truncated Aβ peptides with reduced aggregation propensity and the use of alternative blocking reagents could produce improved outcomes [43]. These modifications may help enhance the distinction between seeded and spontaneous aggregation, thereby increasing assay specificity and sensitivity.

### 4.2 Amplification of α‐Synuclein Seeds

The optimization strategies developed for Aβ_1‐42_ seed amplification were largely applied to α‐synuclein seeds. Amplification of α‐synuclein was considered more promising due to its significantly lower tendency for self‐aggregation, which initiates at 10–15 µM for α‐synuclein compared to 60–120 nM for Aβ_1‐42_. Experiments targeting the amplification of surface‐bound α‐synuclein seeds achieved a detection limit of 0.1–1 nM. While this is a marked improvement compared to Aβ_1‐42_ seeds, it remains insufficient for diagnostic purposes.

When amplification was performed in solution, the detection limit improved significantly, reaching 1 pM. Further refinement of the amplification and detection protocols is expected to push detection limits below 1 pM, potentially enabling much greater sensitivity. Amplification in solution likely performs better due to (1) higher accessibility of binding sites on freely diffusing seeds compared to surface‐immobilized ones, and (2) more efficient diffusion of substrate molecules to the seeds in solution. These factors enhance both the sensitivity and specificity of the assay.

To test the method with natural, biologically relevant samples, an experiment was conducted using surface amplification with brain homogenates. This approach yielded a 3.5‐fold higher signal for homogenates from diseased animals compared to those from healthy controls, demonstrating that natural seed concentrations in animal tissue could be detected. The results for α‐synuclein detection are highly promising. Further optimization efforts using CSF or blood from biobanks could pave the way for the development of a sensitive early diagnostic blood test for PD.

## 5 Conclusions

This study explored seeded aggregation as a detection method for Aβ and α‐synuclein, key biomarkers in AD and PD. While Aβ_1‐42_ exhibited strong self‐aggregation, preventing reliable seed amplification at diagnostic concentrations, α‐synuclein demonstrated significantly lower self‐aggregation, allowing for more sensitive detection. Amplification in solution proved superior to surface‐based methods, achieving a detection limit of 1 pM for α‐synuclein. The approach successfully distinguished diseased from healthy brain homogenates, highlighting its diagnostic potential for PD. Further optimization may enhance sensitivity, potentially enabling early disease detection and facilitating the development of companion diagnostics for neurodegenerative disorders.

## Conflicts of Interest

Oliver Bannach is a cofounder and shareholder of attyloid GmbH. Marlene Pils is an employee of attyloid. Detlev Riesner is a member of attyloid's supervisory board. These factors did not influence the interpretation of the data. The other authors declare no conflicts of interest.

## Supporting information




**Supplementary Materials**: bab70083‐sup‐0001‐SuppMat.pdf

## Data Availability

The data presented in this study are available in the article.
