# Supplementary material for: Analysis of Single Particles of Amyloid Beta and α‐Synuclein With Seeded Amplification for the Diagnosis of Alzheimer's and Parkinson's Disease
Source: Biotechnol Appl Biochem. 2025 Oct 31;73(3):1115–24. doi: 10.1002/bab.70083 (PMC13238379; doi:10.1002/bab.70083)
Supplement: Supplementary file 1 — Supplementary Materials: bab70083‐sup‐0001‐SuppMat.pdf [file BAB-73-1115-s001.pdf]

Analysis of single particles of amyloid beta and α-synuclein with seeded amplification for the diagnosis of Alzheimer's and Parkinson's disease

Alexandra Dybala,[1] Marlene Pils,[2] Oliver Bannach,[2] Gültekin Tamgüney,[1,3,*] Detlev Riesner[1,*]

1 Heinrich-Heine-Universität Düsseldorf, Mathematisch-Naturwissenschaftliche Fakultät, Institut für Physikalische Biologie, Düsseldorf, Germany

2 attyloid GmbH, Düsseldorf, Germany

3 Institute of Biological Information Processing (Structural Biochemistry: IBI-7), Forschungszentrum Jülich, Jülich, Germany

Supplementary Information

Material and Methods

Atomic Force Microscopy

Atomic force microscopy (AFM) was employed to visualize the structural morphology of the seed aggregates. To prepare samples for imaging, the seeds were immobilized in buffer solution onto freshly cleaved mica substrates (AFM Mica Disks, Plano), which belong to the group of phyllosilicates. A volume of 10 µL of Aβ seeds was applied to the mica surface and incubated for 30 minutes in a plastic Petri dish. For Aβ samples, the dish was sealed with its lid, and a moist tissue was placed inside to maintain humidity and prevent drying artifacts. After incubation, the samples were washed three times with 100 µL of ultrapure water to remove unbound material. Excess water was carefully removed using a tissue. The samples were then dried under a gentle stream of gaseous nitrogen. AFM measurements were performed using a JPK NanoWizard III system (Bruker) operated in intermittent contact mode (AC mode). Imaging was carried out with an OMCL-AC160TS cantilever.

Surface plasmon resonance

Experiments were conducted using a Biacore T200 instrument (GE Healthcare) at 25 °C in PBS. A CM5 sensor chip (GE Healthcare) with a dextran matrix was activated with 200 mM 1-(3-dimethylaminopropyl)-3-ethylcarbodiimide hydrochloride (Sigma-Aldrich) and 50 mM N-hydroxysuccinimide (Sigma-Aldrich) for 400 s. Blocking peptides were immobilized at approximately 100 relative units (RU) in 10 mM sodium acetate (pH 5.0).

The sensor surface was deactivated with 1 M ethanolamine (pH 8.5). Capture antibodies were incubated in a cyclic manner with increasing concentrations (0.01-300 nM) under continuous flow conditions. Each cycle included a 5-minute association phase followed by a 15-min dissociation phase, with a flow rate of 30 µL/min. Sensorgrams were analyzed using Biacore T200 Evaluation Software v3.2, employing the bivalent analyte fit model.

Figure S1 Blocking with A$\beta_{1\text{-}14}$ does not effectively prevent spontaneous A$\beta_{1\text{-}42}$ aggregation.

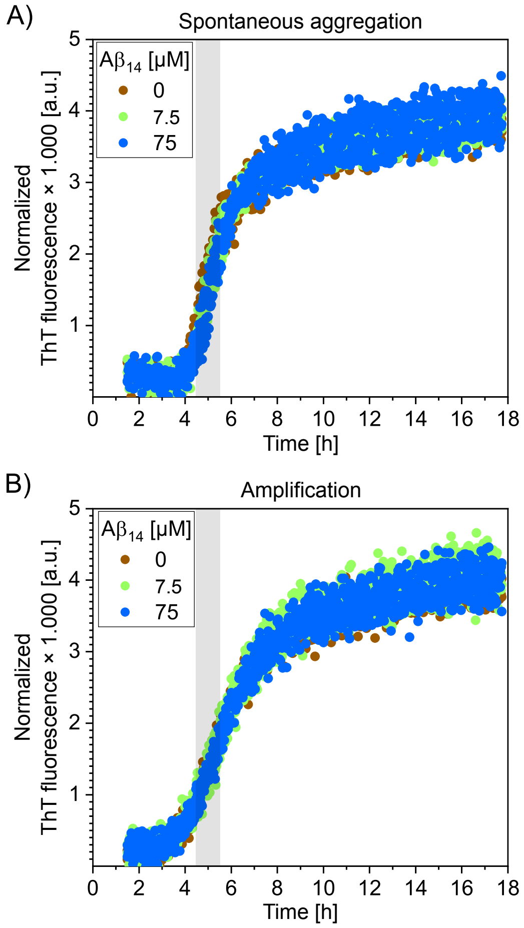

(A and B) The Thioflavin T (ThT) aggregation assay shows the effect of varying concentrations of A$\beta_{1\text{-}14}$ (0 µM, brown; 7.5 µM, green; 75 µM, blue) on the spontaneous aggregation of 1 µM A$\beta_{1\text{-}42}$ monomer substrate (A) or on the amplification of 100 nM A$\beta_{1\text{-}42}$ seeds (B) in phosphate-buffered saline pH 7.4 at room temperature. The data presented are the mean of two replicates. The grey-shaded areas of the graph represent the time period of the linear aggregation phase.

Figure S2 Influence of bovine serum albumin (BSA) on amplification.

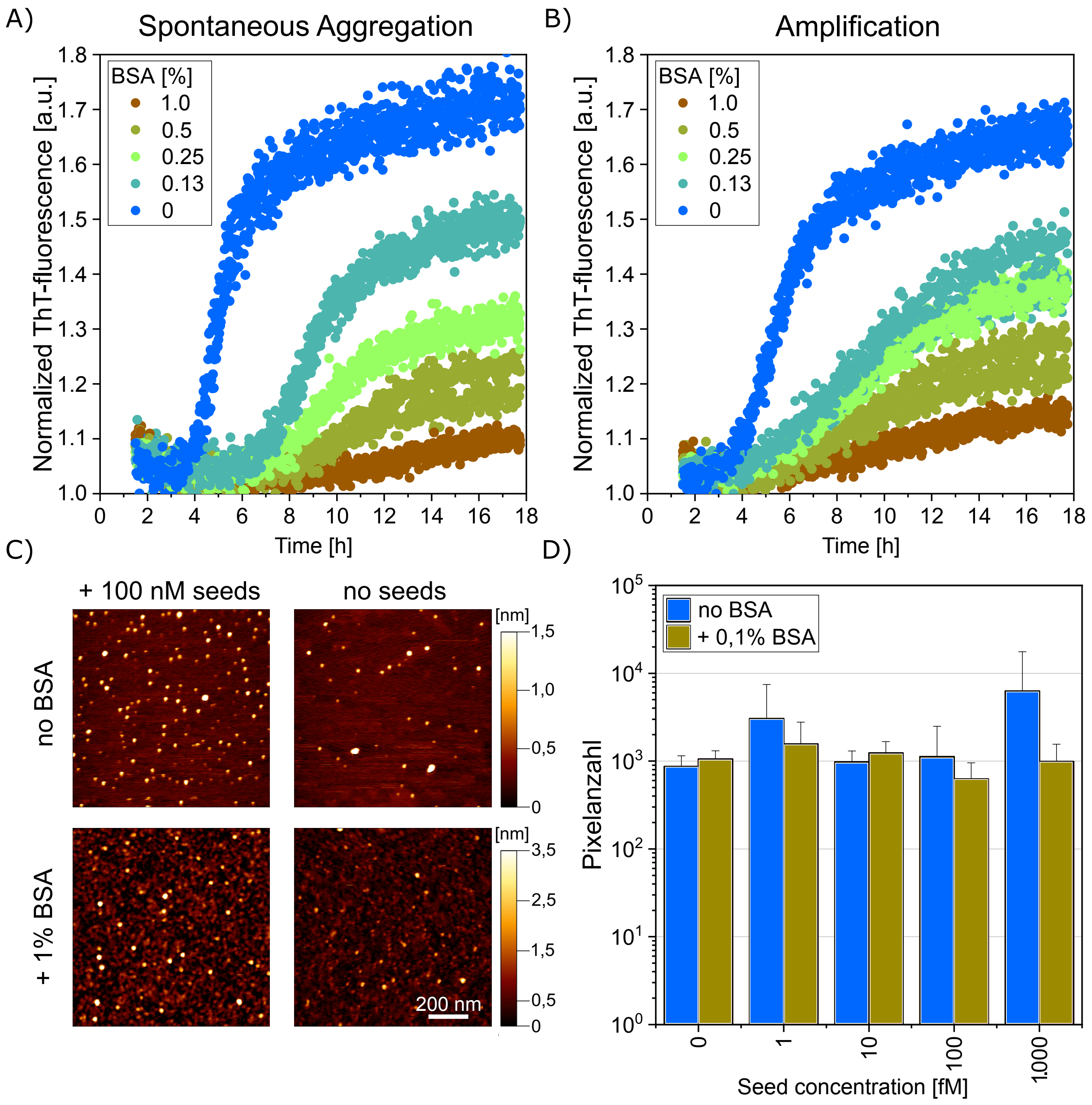

(A and B) Thioflavin T (ThT) fluorescence was employed to monitor the spontaneous aggregation of 1 µM Aβ_{1-42} substrate (A) or amplification by addition of 100 nM seeds in PBS at room temperature (B). The addition of various BSA concentrations ranging from 0.13% to 1% is shown using a color gradient from blue (0% BSA) to brown (1% BSA). Each aggregation curve was normalized to its minimum value after the first 1.5 h were excluded due to the temperature sensitivity of ThT. Number of replicates: n = 2. (C) Atomic force microscopy images of samples with 100 nM seeds or without seeds ±1% BSA. (D) Amplification of 1–1,000 fM Aβ_{1-42} seeds in sFIDA using 100 nM 10% Hilyte-Fluor-647-labeled Aβ_{1-42} substrate ± 0.1% BSA. The capture antibody used was 6E10. The cutoff was set at 0.5% of the seed-free controls in the red fluorescence channel. Amplification was carried out for 20 h at room temperature.

Supplementary Information 1

A$\beta_{11\text{-}42}$ peptide as substrate

The truncated A$\beta_{11\text{-}42}$ peptide lacks the binding epitopes for the antibodies NAB228 and 6E10, preventing its interaction with the capture antibodies immobilized on the surface. Instead, it is expected to bind A$\beta_{1\text{-}42}$ seeds and facilitate their amplification. This behavior was evaluated using micromolar concentrations of A$\beta_{11\text{-}42}$ as the substrate and 100 nM A$\beta_{1\text{-}42}$ as seeds. Amplification was observed within the first hour of incubation, whereas self-aggregation of A$\beta_{11\text{-}42}$ occurred after 1 h at 5 µM and after 5 h at 1 µM. However, to detect picomolar or lower seed concentrations using 0.1 or 1 µM A$\beta_{11\text{-}42}$ substrate, the amplification time was extended to 24 h at room temperature. Under these diagnostic-like conditions, no significant amplification effect was observed. It is likely that nonspecific binding of A$\beta_{11\text{-}42}$ aggregates to the surface obscured the specific, seed-dependent signal.
